# Heterogeneous distribution of tau pathology in the behavioural variant of Alzheimer’s disease

**DOI:** 10.1136/jnnp-2020-325497

**Published:** 2021-04-13

**Authors:** Ellen Singleton, Oskar Hansson, Yolande A. L. Pijnenburg, Renaud La Joie, William G Mantyh, Pontus Tideman, Erik Stomrud, Antoine Leuzy, Maurits Johansson, Olof Strandberg, Ruben Smith, Evi Berendrecht, Bruce L Miller, Leonardo Iaccarino, Lauren Edwards, Amelia Strom, Emma E Wolters, Emma Coomans, Denise Visser, Sandeep S V Golla, Hayel Tuncel, Femke Bouwman, John Cornelis Van Swieten, Janne M Papma, Bart van Berckel, Philip Scheltens, Anke A. Dijkstra, Gil D Rabinovici, Rik Ossenkoppele

**Affiliations:** 1 Alzheimer Center Amsterdam, Department of Neurology, Amsterdam Neuroscience, Vrije universiteit Amsterdam, Amsterdam UMC, Amsterdam, the Netherlands; 2 Clinical Memory Research Unit, Department of Clinical Sciences Malmö, Lund University, Lund, Sweden; 3 Memory and Aging Center, Department of Neurology, Weill Institute for Neurosciences, University of California, San Francisco, San Francisco, California, USA; 4 Memory Clinic, Skåne University Hospital Lund, Lund, Sweden; 5 In Vivo Human Molecular and Structural Neuroimaging Unit, Division of Neuroscience, IRCCS San Raffaele Scientific Institute, Milan, Italy; 6 Department of Radiology and Nuclear Medicine, Amsterdam Neuroscience, Vrije universiteit Amsterdam, Amsterdam UMC, Amsterdam, the Netherlands; 7 Department of Neurology, Erasmus Medical Center, Rotterdam, the Netherlands; 8 Department of Pathology, Amsterdam Neuroscience, Vrije universiteit Amsterdam, Amsterdam UMC, Amsterdam, the Netherlands

## Abstract

**Objective:**

The clinical phenotype of the rare behavioural variant of Alzheimer’s disease (bvAD) is insufficiently understood. Given the strong clinico-anatomical correlations of tau pathology in AD, we investigated the distribution of tau deposits in bvAD, in-vivo and ex-vivo, using positron emission tomography (PET) and postmortem examination.

**Methods:**

For the tau PET study, seven amyloid-β positive bvAD patients underwent [^18^F]flortaucipir or [^18^F]RO948 PET. We converted tau PET uptake values into standardised (W-)scores, adjusting for age, sex and mini mental state examination in a ‘typical’ memory-predominant AD (n=205) group. W-scores were computed within entorhinal, temporoparietal, medial and lateral prefrontal, insular and whole-brain regions-of-interest, frontal-to-entorhinal and frontal-to-parietal ratios and within intrinsic functional connectivity network templates. For the postmortem study, the percentage of AT8 (tau)-positive area in hippocampus CA1, temporal, parietal, frontal and insular cortices were compared between autopsy-confirmed patients with bvAD (n=8) and typical AD (tAD;n=7).

**Results:**

Individual regional W-scores ≥1.96 (corresponding to p<0.05) were observed in three cases, that is, case #5: medial prefrontal cortex (W=2.13) and anterior default mode network (W=3.79), case #2: lateral prefrontal cortex (W=2.79) and salience network (W=2.77), and case #7: frontal-to-entorhinal ratio (W=2.04). The remaining four cases fell within the normal distributions of the tAD group. Postmortem AT8 staining indicated no group-level regional differences in phosphorylated tau levels between bvAD and tAD (all p>0.05).

**Conclusions:**

Both in-vivo and ex-vivo, patients with bvAD showed heterogeneous distributions of tau pathology. Since key regions involved in behavioural regulation were not consistently disproportionally affected by tau pathology, other factors are more likely driving the clinical phenotype in bvAD.

## Introduction

Individuals with the behavioural variant of Alzheimer’s disease (bvAD, previously referred to as ‘frontal AD’) experience early prominent behavioural symptoms and personality changes, such as disinhibition, compulsive behaviours and loss of empathy.^1 2^ These individuals are clinically reminiscent of behavioural variant frontotemporal dementia (bvFTD), but have AD as primary pathology and resemble patients with ‘typical’ AD (tAD) neuroanatomically, as atrophy and hypometabolic patterns in bvAD predominantly occur in temporoparietal regions.^1 3^ Imaging and pathological investigations (mostly case reports or small cohort studies based on the low prevalence of this phenotype^1^) have provided mixed results regarding the involvement of the frontal cortex in bvAD.^4–8^ This apparent clinico-anatomical dissociation indicates the need for a better understanding of the neurobiological factors underlying the bvAD phenotype. To that end, it is crucial to study the distribution of tau deposition in bvAD, as this central neuropathological hallmark of AD is closely related to type and severity of cognitive symptoms^9^ and precedes and predicts patterns of neurodegeneration detected by MRI and [^18^F]-fluorodeoxyglucose (FDG) positron emission tomography (PET).^10 11^ In this study, we aimed to investigate the regional distribution of tau pathology in bvAD (i) in-vivo using tau PET and (ii) ex-vivo using postmortem examination.

## Methods

### Participants

For the tau PET study, we included seven patients clinically diagnosed with bvAD from the Amsterdam Dementia Cohort (ADC, the Netherlands, n=2), the University of California San Francisco (UCSF, USA, n=3) Alzheimer Disease Research Center and the Swedish BioFINDER study (http://www.biofinder.se; Sweden, n=2). In the absence of formal clinical consensus criteria for bvAD we used our previously established procedure.^1^ First, among participants with available tau PET, we selected those with a clinical diagnosis of AD dementia^12^ or mild cognitive impairment (MCI).^13^ Second, from this selection we included only patients who were on the AD pathological continuum according to the National Institute on Aging and Alzheimer’s Association (NIA-AA) research criteria^10^ of amyloid-β positivity based on PET or cerebrospinal fluid (CSF). Third, we performed extensive chart reviews (by RO) and included only participants fulfilling ≥2 of 6 core clinical criteria for bvFTD,^14^ consisting of apathy, loss of empathy, disinhibition, compulsive behaviours, hyperorality and dysexecutive functioning. This ensured the inclusion of patients with robust and clinically prominent ‘bvFTD-like’ symptoms, and was based on our previous finding that 75% of bvAD patients showed ≥2 bvFTD clinical symptoms, and bvAD patients generally showed a slightly milder behavioural profile than patients with bvFTD.^1^ We quantified the degree of behavioural impairment in the current study using the Neuropsychiatric Inventory (NPI)^15^ at the ADC, the Mild Behavioral Impairment (MBI-C)^16^ for the BioFINDER study and the Affect Naming Task,^17^ a social cognition test assessing emotion recognition, at UCSF. Note that we excluded participants with dysexecutive AD^18^ if they did not meet any of the remaining five bvFTD criteria, in order to selectively study the above-mentioned core behavioural features in AD. None of the patients with bvAD in the PET study were included in our prior work. We compared the participants with bvAD to participants with tAD from all centres (ADC, n=55; UCSF, n=60; BioFINDER, n=90), consisting of Aβ-positive AD dementia and MCI participants who had undergone tau PET. Participants meeting diagnostic criteria for posterior cortical atrophy or the logopenic variant of primary progressive aphasia were excluded from this group. In addition, patients with known autosomal dominant mutations for AD or FTD were excluded. A clinical description of the bvAD cases can be found in online supplemental table 1. For the postmortem study, eight patients clinically diagnosed with bvAD who donated their brains to the Netherlands Brain Bank were compared with seven participants with tAD. These diagnoses were established retrospectively based on antemortem clinical diagnosis of ‘frontal variant of AD’, bvFTD or a differential diagnosis of bvFTD versus AD.^1^ All patients with bvAD and tAD had a primary neuropathological diagnosis of AD.

10.1136/jnnp-2020-325497.supp1 Supplementary data

### Tau PET in bvAD compared with tAD

PET scanning was performed using the tau tracers [^18^F]flortaucipir (ADC, UCSF) and [^18^F]RO948 (BioFINDER). Image acquisition and processing for each centre have been described previously^9 19 20^ and are summarised in online supplemental table 2. Briefly, we generated standardised uptake value ratios (SUVR) for the interval between 80 and 100 ([^18^F]flortaucipir) or 70–90 ([^18^F]RO948) minutes post-injection using (inferior) cerebellar grey cortex as the reference region. We then computed native space derived mean SUVR values in the following (composite) regions-of-interests (ROIs) representing a mix of AD and bvFTD vulnerable regions: entorhinal, temporoparietal, frontal, and insular cortices, and whole cortex. To examine the relative tau burden in frontal regions compared with classical AD regions, we additionally computed frontal-to-entorhinal and frontal-to-parietal ratios. A detailed composition of each ROI is shown in online supplemental table 3. Furthermore, mean SUVR values were extracted from four functional connectivity network templates in Montreal Neurological Institute (MNI) space implicated in AD and bvFTD, including the executive control network, salience network, anterior default mode network and posterior default mode network.^21^ For each ROI we computed W-scores reflecting standardised individual differences between the observed and predicted SUVR based on the tAD distribution, adjusted for age, sex and mini mental state examination (MMSE) score (ie, W=(observed SUVR–predicted SUVR)/SDresiduals)). Note that the limited sample size and differences in tau PET acquisition across cohorts did not allow group-wise statistical comparisons, hence, results are described as the W-score in individual patients with bvAD relative to the normal distribution across the tAD group (ie, W scores≥ 1.96, corresponding to p<0.05). For visual purposes, the coregistered T1-weighted MRI scans were warped to Montreal Neurological Institute (MNI152) space, and these transformation matrixes were applied to warp native space SUVR images to MNI space. The normalised PET images were then smoothed using an 8 mm Gaussian kernel. The tau PET images of individual patients with bvAD were visually compared with an average SUVR image for the (cohort-specific) tAD groups.

### Associations between tau PET patterns and age in bvAD relative to tAD

We then examined the influence of age-of-onset on the involvement of the frontal regions in bvAD, as younger age has previously been linked to greater tau pathology across the neocortex.^22^ Therefore, the associations between age and tau PET uptake in medial prefrontal, lateral prefrontal, salience network and anterior default mode network regions were plotted and the tau PET SUVRs of the bvAD cases were studied relative to the distribution of the tAD groups.

### Postmortem investigation of tau pathology in bvAD compared with tAD

Immunohistochemistry was performed with antibodies against phosphorylated tau using AT8 (AT8 antibody, 1:800 dilution, ThermoFisher, Waltham, USA) on 8 µm thick representative sections of the anterior cingulate cortex, hippocampus CA1, caudate nucleus, entorhinal cortex, frontal pole, frontoinsula, putamen, subiculum and thalamus of the right hemisphere. A detailed description of the procedures can be found in online supplemental table 4. The presence of chromogen 3.3′-diaminobenzidine (DAB: K5007; DAKO) staining was quantified using the colour threshold plugin in ImageJ (V.1.52u; NIH), where the threshold was set to include tangles and threads. Of each region, two images were taken and the outcome measurement was the average percentage of DAB-stained pixels per brain region. Systematic staining was performed for Aβ42, α-synuclein and 3R and 4R tau and TDP-43. Between group differences in percentage of tau pathology brain region were assessed using Mann-Whitney U tests, adjusting for age and sex. We used R V.4.0.2 (https://www.R-project.org/) for statistical analyses. A p value below 0.05 was considered significant.

## Results

### Demographic characteristics participants with bvAD

Demographic and clinical characteristics of the participants are presented in tables 1 and 2. In the tau PET study, 6/7 (85.7%) bvAD cases were male, while 48.3% of patients with tAD were male. Age ranged from 59 to 80 in the bvAD cases (mean: 69.1±8.4), compared with a mean age of 67.8±7.7 in the tAD groups. MMSE ranged between 17 and 26 in bvAD cases (mean: 21.7±2.8), with average MMSE scores of 21.8±4.8 in the tAD cases (table 1). 3/7 bvAD cases were *APOE* ε4 positive, 3/7 *APOE* ε3 homozygote and *APOE* genotype was missing for 1 bvAD case, while *APOE* ε4 positivity was found in 70% of the tAD cases. Presence of bvFTD symptoms (maximum is 6) ranged from 2 to 6 in bvAD cases, with apathy as the most prevalent symptom (n=6), followed by disinhibition (n=5), loss of empathy, compulsiveness and hyperorality (all n=3), and dysexecutive profile (n=1). Scores on the NPI were 20 in bvAD case #1 and 41 in case #2 compared with a median [interquartile] of 7 [10] in tAD (n=29). The MBI-C score was missing for bvAD case #6 and was 18 in case #7 compared with a median [interquartile] of 11 [15] in tAD (n=50). The affect naming z-scores were −2.47 in bvAD case #3, –1.88 in case #4 and −0.40 in case #5 compared with a mean of −0.41±1.60 in tAD (n=59). In the postmortem study, 4/8 (50.0%) bvAD cases were male versus 3/7 (42.9%) in the tAD group, and the mean age at death was 66.6±6.0 in the bvAD group versus a mean age of 69.1±3.3 in the tAD group (table 2). Disease duration was slightly longer in bvAD cases (6.3±3.6 years) compared with tAD cases (4.6±3.3 years).

**Table 1:** Characteristics of participants with the behavioural variant of Alzheimer’s disease (presented individually) and participants with typical Alzheimer’s disease (presented as a group) in the positron emission tomography study

	ADC			UCSF				BioFINDER		
	bvAD_1_	bvAD_2_	Typical AD	bvAD_3_	bvAD_4_	bvAD_5_	Typical AD	bvAD_6_	bvAD_7_	Typical AD
n			55				60			90
Age	73	62	65.7 (7.7)	59	60	80	64.5 (8.8)	80	70	73.1 (6.6)
Sex (% male)	m	m	49%	m	m	m	42%	f	m	54%
Education (years)	13	13	12.2 (3.1)^a^	16	16	19	17.3 (3.1)^c^	7	9	12.2 (4.9)^f^
MMSE	17	22	23.2 (3.9)	24	21	19	22.1 (6.5)^d^	26	23	20.2 (4.1)^g^
APOE (% ε4 pos.)	ε3ε3	ε3ε3	79%^b^	ε3ε3	ε3ε4	–	58%^e^	ε3ε4	ε3ε4	73%^h^
bvFTD criteria										
Disinhibition	Y	N		Y	Y	N		Y	Y	
Apathy	Y	Y		Y	N	Y		Y	Y	
Loss of empathy	Y	N		N	N	Y		Y	N	
Compulsiveness	N	Y		Y	Y	N		N	N	
Hyperorality	Y	Y		N	N	N		N	Y	
Dysexecutive	N	N		N	N	N		Y	N	
n	4	3		3	2	2		4	3	
Tau SUVR										
Entorhinal	1.27	1.81	1.49 (0.25)	1.53	1.49	1.89	1.73 (0.35)	1.59	1.91	1.98 (0.42)
Temporoparietal	1.56	1.80	1.58 (0.40)	1.67	1.74	1.76	1.94 (0.56)	1.40	2.28	1.84 (0.61)
Medial frontal	1.45	1.53	1.25 (0.28)	1.39	1.50	2.05	1.48 (0.35)	1.15	1.77	1.38 (0.54)
Lateral frontal	1.58	2.21	1.39 (0.38)	1.43	1.69	1.88	1.68 (0.50)	1.15	2.28	1.46 (0.63)
Insula	1.43	1.24	1.22 (0.15)	1.12	1.14	1.35	1.22 (0.14)	1.06	1.12	1.19 (0.21)
Mean cortical	1.51	1.62	1.44 (0.30)	1.53	1.58	1.82	1.72 (0.43)	1.30	2.10	1.66 (0.51)
Frontal:Entorhinal	1.19	0.99	0.89 (0.21)	0.92	1.07	1.04	0.92 (0.21)	0.78	1.16	0.78 (0.22)
Frontal:Parietal	1.04	0.92	0.84 (0.14)	0.79	0.89	1.18	0.83 (0.16)	0.97	0.96	0.90 (0.20)

*n=51 for education ADC.

†n=48 for education UCSF.

‡n=86 for education BioFINDER.

§n=50 for MMSE UCSF.

¶n=89 for MMSE BioFINDER.

**n=47 for *APOE* ε4 ADC.

††n=43 for *APOE* ε4 UCSF.

‡‡n=88 for *APOE* ε4 BioFINDER.

ADC, Amsterdam Dementia Cohort; bvAD, behavioural variant of Alzheimer’s disease; bvFTD, behavioural variant frontotemporal dementia; MMSE, mini mental state examination; SUVR, standardised uptake value ratios; UCSF, University of California San Francisco.

**Table 2:** Characteristics of participants with behavioural variant of Alzheimer’s disease and typical Alzheimer’s disease in the postmortem study

Case	Dx	Sex	Age at death	Disease duration (months)	Dx neuro-path.	ABC score	Brain weight (g)	Cause of death	PMI (hours)	CAA	CVD	LB	TDP	ARTAG
1	bvAD	F	58	34	AD	A3 B2 C3	1127	Dehydration	5.25	Absent	Absent	Absent	Absent	Absent
2	bvAD	F	75	13	AD	A3 B3 C3	1230	Cardiac arrest	15.0	Absent	Unknown	Absent	Absent	Present
3	bvAD	M	58	57	AD	A3 B3 C3	1195	Cachexia	5.25	Present	Present	Amyg & subst. nigra	Absent	Present
4	bvAD	M	74	63	AD	A3 B3 C3	1230	Cachexia	3.42	Present	Absent	Amygdala	Absent	Present
5	bvAD	M	67	57	AD	A3 B3 C3	1263	Euthanasia	8.33	Absent	Present	Amygdala	Absent	Present
6	bvAD	M	67	130	AD+ DLB	A3 B3 C3	973	Epilepsy	3.75	Present	Present	Limbic	Absent	Present
7	bvAD	F	64	104	AD	A3 B3 C3	860	Pneumonia	7.17	Present	Present	Amygdala	Absent	Present
8	bvAD	F	70	143	AD	A3 B3 C3	870	Sepsis	4.00	Present	Absent	Entorhinal	Absent	Present
***M***		**50% m**	**66.6 (6.0)**	**75.1 (43.0)**			**1093.5 (156.7)**		**6.52 (3.58)**	**5**	**4**	**6**	**0**	**7**
9	tAD	M	74	n/a	AD	A3 B3 C3	930	Pneumonia	5.17	Present	Absent	Absent	Absent	Present
10	tAD	F	66	70	AD	A3 B3 C3	915	Pneumonia, cachexia	4.20	Absent	Absent	Amygdala	Absent	Present
11	tAD	F	68	125	AD	A3 B3 C3	835	Dehydration	5.00	Present	Present	Amygdala & temporal	Hip & amyg (LATE-NC)	Present
12	tAD	M	70	10	AD	A3 B3 C3	1400	Cachexia, dehydration	3.17	Present	Present	Absent	Hip & amyg (LATE-NC)	Present
13	tAD	F	73	≥36	AD	A3 B3 C3	970	Cachexia	4.17	Present	Absent	Amygdala	Absent	Present
14	tAD	F	69	34	AD	A3 B3 C3	1065	Urosepsis	6.17	Present	Present	Absent	Absent	Present
15	tAD	F	64	288	AD	A3 B3 C3	760	Cachexia	6.50	Absent	Present	Amygdala	Hip & amyg (LATE-NC)	Present
***M***		**29% m**	**69.1 (3.3)**	**93.8 (94.2)**			**982.1 (180.2)**		**4.91 (1.09)**	**5**	**4**	**4**	**3**	**7**

ABC score, amyloid, Braak CERAD criteria; AD, Alzheimer’s disease; ARTAG, aging-related tau astrogliopathy; bvAD, behavioral variant Alzheimer’s disease; CAA, cerebral amyloid angiopathy; CVD, cerebral vascular disease; DLB, dementia with Lewy bodies; F, female; LB, Lewy bodies; M, male; MMSE, mini mental state examination; n/a or, not available; PMI, postmortem interval in hours; tAD, typical Alzheimer’s disease; TDP, TAR DNA binding protein.

### Tau PET in bvAD compared with tAD

**Figure 1:**
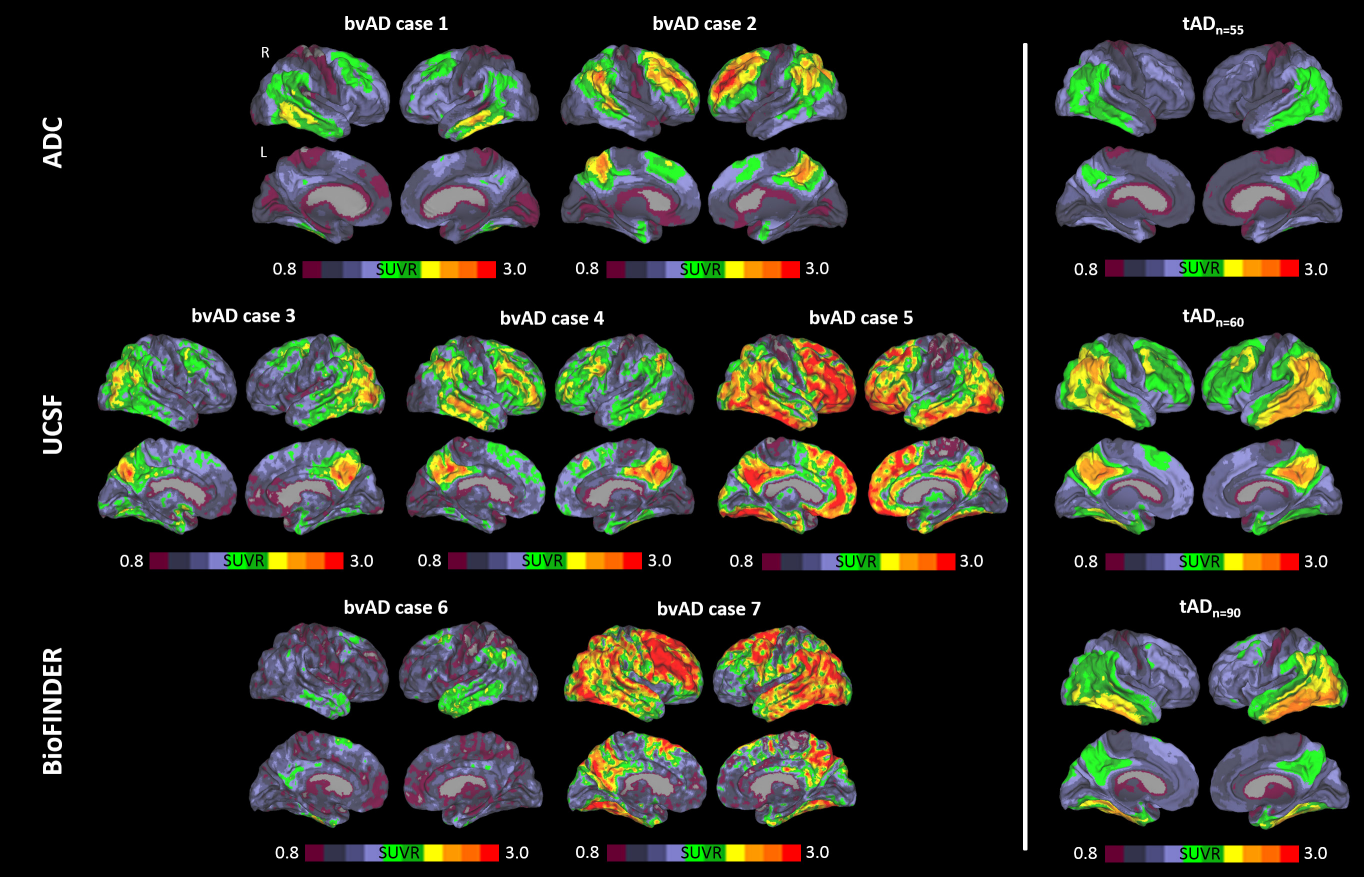
Distribution of tau pathology across the brain of participants with the behavioural variant of Alzheimer’s disease (bvAD, displayed individually) versus participants with the typical Alzheimer’s disease (tAD, displayed as the average of the group). ADC, Amsterdam Dementia Cohort; SUVR, standardised uptake value ratio; UCSF, University of California San Francisco.

**Figure 2:**
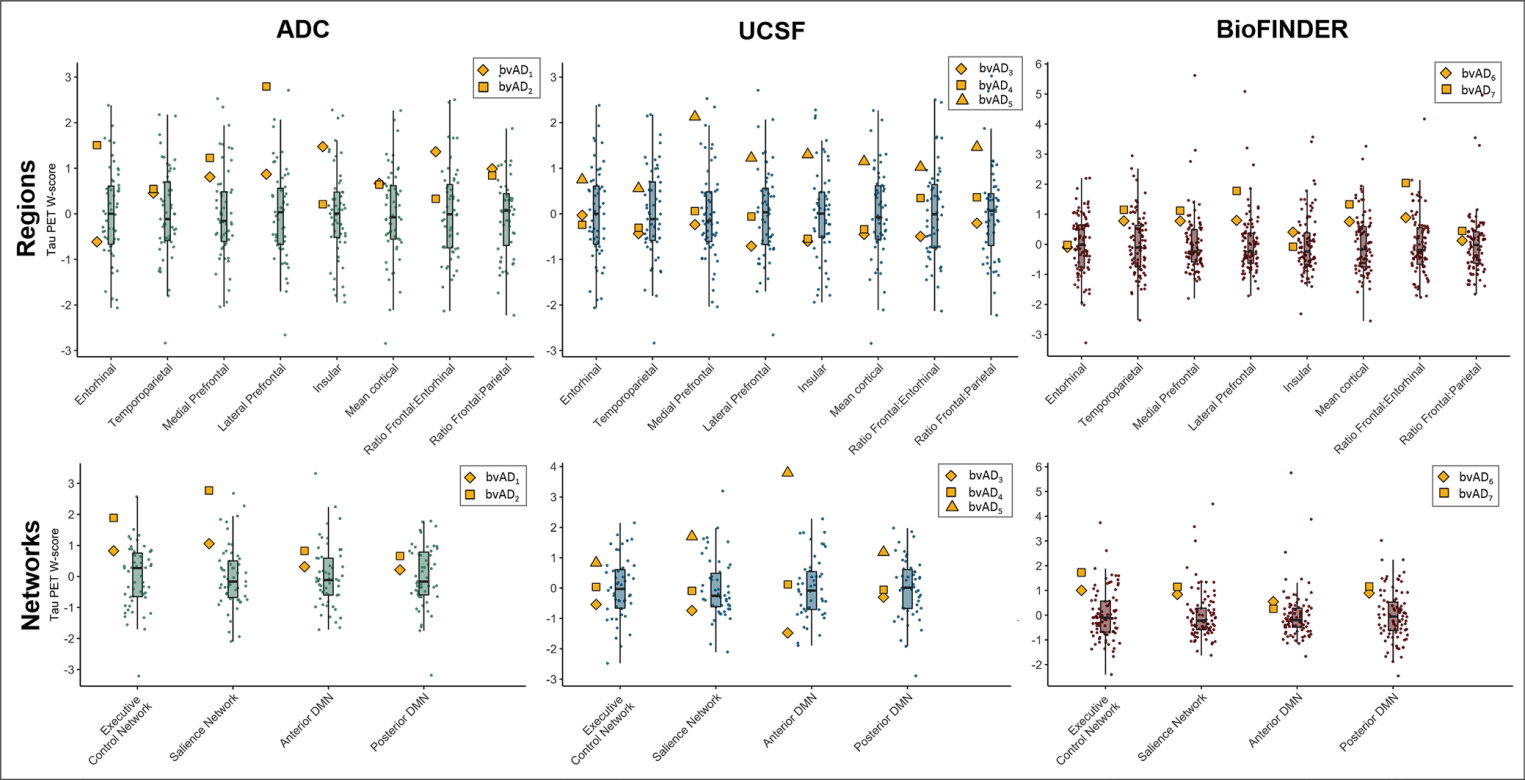
Regional tau positron emission tomography retention in participants with bvAD relative to the distribution of participants with typical AD (tAD). The yellow symbols represent the individual bvAD cases and the boxplots and raincloud plots represent the distributions of the tAD groups. AD, Alzheimer’s disease; ADC, Amsterdam Dementia Cohort; UCSF, University of California San Francisco.

Figure 1 shows the tau PET patterns for all individual bvAD cases relative to an average tau PET image for the whole tAD group per cohort. Visual assessment indicated that 3/7 bvAD cases (#2, #5 and #7) showed prominent frontal involvement in addition to substantial temporoparietal uptake. Among these cases, case #5 showed strongly elevated uptake in the medial prefrontal cortex, while #2 and #7 showed predominant lateral frontal uptake. One case (#4) showed some uptake in the lateral frontal cortex, but the medial parietal cortex was clearly the most affected brain region. 2/7 cases (#1 and #6) had a lateral temporal predominant uptake pattern with very limited frontal involvement. One case (#3) showed a classical AD-like temporoparietal uptake pattern with minimal tracer retention in the frontal cortex. The heterogeneity in tau patterns across patients with bvAD was confirmed by quantitative ROI analyses, showing W-scores ≥1.96 only in one case in the medial prefrontal (#5, W=2.13) and lateral prefrontal (#2, W=2.79) regions and in one case in the ratio frontal-to-entorhinal tau (#7, W=2.04; online supplemental table 5 and figure 2). All ROIs and ratios in the remaining four cases fell within the normal distribution of the tAD group. Regarding tau uptake within functional connectivity network templates, W-scores ≥1.96 were found in one case (#2, W=2.77) in the salience network and another case (#5, W=3.79) in the anterior default mode network (online supplemental tables 6 and 7 and figure 2). All network W-scores in the remaining five cases fell within the normal distribution of the tAD group.

### Associations between tau PET patterns and age in bvAD relative to tAD

**Figure 3:**
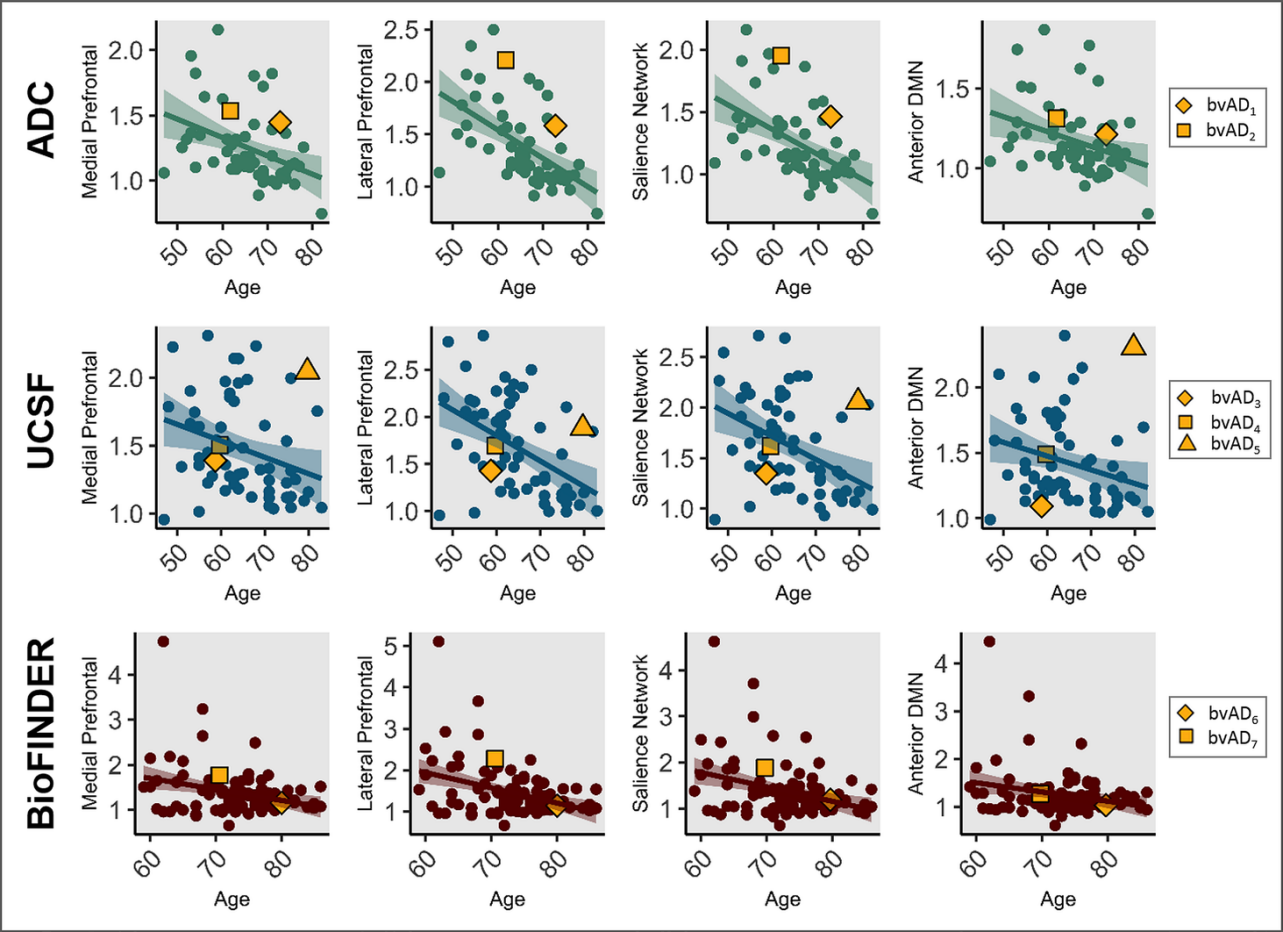
Scatterplots depicting the relationship between frontal, medial prefrontal and lateral prefrontal tau uptake and age in typical Alzheimer’s disease and bvAD across centres. ADC, Amsterdam Dementia Cohort; bvAD, behavioural variant of Alzheimer’s disease; UCSF, University of California San Francisco; DMN, Default Mode Network.

Among the three early-onset (<65 years) bvAD cases, (lateral) frontal tau PET uptake was evident in case #2, moderate in case #4 and limited in case #3 (figure 1). The late-onset bvAD cases were characterised by prominent frontal tau PET uptake in cases #5 and #7 and relative frontal sparing in cases #1 and #6. The heterogeneity of frontal involvement across the age span was further supported by the assessment tau PET uptake in four relevant brain regions/networks (figure 3). This analysis showed that three bvAD cases (#2, #3 and #5) showed substantial higher tau PET uptake than estimated based on their age in the tAD group (observed data exceeded the 95% CI), while tau PET uptake in the remaining four bvAD cases largely overlapped with the 95% CI of the tAD group.

**Figure 4:**
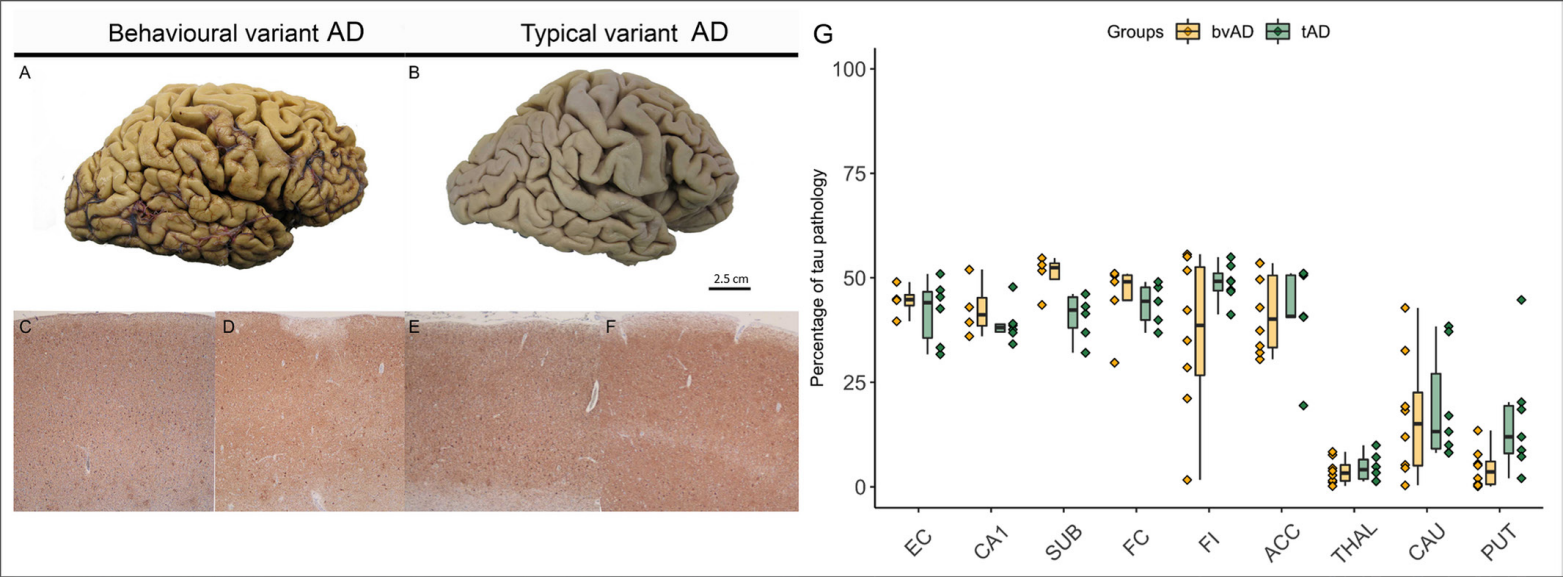
Postmortem tau immunohistochemistry in patients with bvAD and typical AD (tAD). (A) and (B) show images of postmortem brain tissue of a representative case of bvAD (A) and tAD (B), showing similar morphology. The frontal cortices are depicted in (C) and (E) and the entorhinal cortices are depicted in (D) and (F). These images suggest that the tau burden in frontal regions in bvAD do not differ substantially from the burden in tAD, and that the tau burdens between frontal and entorhinal cortices in both bvAD and tAD do not differ from each other. (G) The percentage of tau pathology in regions of interest in participants with bvAD and participants with tAD, showing no significant differences between the two groups. ACC, anterior cingulate cortex; AD, Alzheimer’s disease; bvAD, behavioural variant of Alzheimer’s disease; CAU, caudate nucleus; CA1, hippocampus CA1; EC, entorhinal cortex; FC, frontal cortex; FI, frontoinsula; PUT, putamen; SUB, subiculum; THAL, thalamus.

### Postmortem investigation of tau pathology in bvAD compared with tAD

Presence of tau pathology quantified using AT8 immunohistochemistry did not show significant differences between bvAD and tAD groups in any of the investigated brain regions (all p>0.05; figure 4 and online supplemental table 8). One bvAD case had Lewy body disease as coprimary neuropathological diagnosis in addition to AD. In terms of comorbid pathologies, Lewy body pathology was present in 6/8 patients with bvAD versus 4/7 patients with tAD. Cerebral amyloid angiopathy (CAA) was found in 5/8 bvAD cases and in 5/7 tAD cases. Cerebral vascular disease was found in 4/7 bvAD cases and in 4/7 tAD cases. All bvAD cases were negative for TDP-43, while 3/7 tAD patient showed TDP-43 inclusions in the hippocampus and amygdala, reflecting LATE-NC stage 2. In addition, in none of the bvAD cases the presence of 3R tau was observed in isolation.

## Discussion

In this multicentre case series, we examined the distribution of tau pathology based on PET and postmortem evaluations in clinically defined and amyloid-β positive individuals with bvAD. We found a heterogeneous distribution of tau pathology across individual participants with bvAD, ranging from pronounced anterior involvement to a more temporoparietal pattern based on PET. Group-level immunohistochemistry in an independent sample of patients with bvAD supported this heterogeneous distribution of hyperphosphorylated tau pathology across different brain regions, which did not differ from the distribution in tAD. Altogether, as frontal regions were not consistently disproportionally affected by in-vivo and ex-vivo tau pathology in these patients with bvFTD-like phenotypes, these results suggests that tau pathology may not be the main or sole driver of the clinical phenotype in bvAD.

Our results corroborate the remarkable heterogeneity of tau distributions described in the scarce neuropathological and PET literature on bvAD. While some neuropathological studies indicate more pronounced tau pathology in frontal regions than in other brain regions,^6 23 24^ others describe a widespread distribution of tau across different lobes in bvAD participants^25–27^ or no differences in the burden of frontal tau pathology in bvAD compared with tAD.^4^ Neuropathological studies typically lack the ability to make inferences on the distributions of tau in early stages of the disease, which is a major advantage of neuroimaging techniques like PET. However, the few in-vivo investigations of tau PET in bvAD to date have shown somewhat contradictory results. While one case study suggested frontal involvement in addition to a temporoparietal pattern in a bvAD case with advanced dementia (MMSE: 10/30),^27^ another bvAD case with mild dementia (MMSE: 21/30) showed a predominant temporoparietal pattern of tracer retention with sparing of frontal regions.^9^ Furthermore, a group study (n=15) combining cases with behavioural and dysexecutive AD suggested frontal involvement of tau pathology measured with PET, in the absence of marked frontal brain atrophy.^28^ Our extended case series shows that patients with bvAD are primarily characterised by a classical temporoparietal pattern of tau, with, in some cases, pronounced involvement of (mostly lateral) frontal areas, which did not strongly depend on disease severity or age of onset. Importantly, most bvAD cases did not show prominent tau uptake in medial prefrontal and insular regions, which are affected in bvFTD and constitute key regions of the salience network^29^ that regulates complex social behaviours. Indeed, only one case showed disproportionate tau deposition in the medial prefrontal cortex and salience network relative to other brain regions. This is in contrast to other atypical AD variants which almost invariably show tau PET patterns that correspond to their clinical phenotype, that is, predominant occipito-temporal and/or occipito-parietal involvement in posterior cortical atrophy (the ‘visual’ variant of AD) or highly asymmetric (left>right) tau PET uptake in language network regions in logopenic variant primary progressive aphasia (the ‘language’ variant of AD).^9 30^ A possible explanation for the discrepancy in bvAD could be that behavioural and socio-emotional processing entail more multifaceted constructs than neurocognitive domains like language and visual functions, and therefore engage wider (sub)cortical regions and networks across the brain.^31^

Besides tau pathology, several other mechanisms may underlie the clinical phenotype in bvAD. First, pathologies other than AD may be driving the behavioural abnormalities. For example, co-occurrence of Lewy body pathology has been observed in more than half of patients with a clinical diagnosis of bvFTD who were neuropathologically diagnosed with AD.^32^ However, in our study only one case had a coprimary neuropathological diagnosis of dementia with Lewy bodies in addition to AD, and this low frequency is in accordance with previous pathological findings in clinically defined bvAD.^1^ Importantly, TDP-43 or isolated 3R tau inclusions were not found in our bvAD cases. As substantial CAA and comorbid Lewy body inclusions were found in both our bvAD and tAD patients, these comorbid pathologies are likely not driving the differences in clinical phenotypes. Second, patients with bvAD may show lower density of Von Economo neurons (VENs). VENs are large bipolar projection neurons located exclusively in the anterior cingulate cortex and the frontoinsula^33^ that are affected in bvFTD and psychiatric diseases and are implicated in higher-order social functioning and thus crucial to adaptive behavioural regulation. No significant difference in VEN density was observed in the anterior cingulate cortex between bvAD cases and tAD cases in a sample of donors with coexisting Lewy body pathology,^34^ leaving the role of the VENs in ‘pure’ bvAD unknown. Third, the behavioural disturbances observed in bvAD may arise from damage to deep grey matter or white matter structures that have previously been linked to neuropsychiatric symptoms,^35 36^ rather than from frontal neocortical pathology. However, except for the amygdala, we previously observed no differences in grey matter volumes or patterns of white matter hyperintensities between bvAD and tAD that are of relevance for behaviour.^3^ In addition, the current study showed no differences in postmortem tau pathology in subcortical regions between bvAD and tAD, supporting the notion that these structures may not be disproportionally affected in bvAD. Alternatively, the explanation may lie in functional rather than structural mechanisms, as behaviour may rely on complex integrated networks across the brain and we previously showed alterations in metabolic connectivity of the anterior default mode network in bvAD.^3^ In addition, analogous to reports of participants with the logopenic variant of progressive aphasia showing learning disabilities in their medical history,^37^ the presence of premorbid vulnerable personality structures in participants with bvAD—or a pathological interplay between personality traits and AD pathology^38^—may provide clues to understand the clinical phenotype in bvAD. It is conceivable that these vulnerable personality structures are exacerbated once AD pathological changes start to affect the brain, independent of the precise anatomical localisation of protein deposition. Future studies should examine this hypothesis and should also include an assessment of sex differences given the male predominance in bvAD.

Strengths of the current study include the relatively large sample of amyloid-β positive bvAD cases who met ≥2/6 bvFTD clinical criteria and underwent tau PET or autopsy. In addition, the comparison to cohort-specific reference groups of tAD patients aids the clinical interpretation of our findings. Limitations of this study mainly lie in the descriptive nature of in particular the tau PET study, as statistical comparisons were hampered by the small sample size in the bvAD cases due to the low prevalence of this clinical phenotype. In addition, different tau tracers and PET processing pipelines were applied at the different centres, hampering pooling of tau PET data. Second, the presence of comorbid pathology contributing to the clinical presentation cannot be excluded in the tau PET study. Third, ideally autopsy and tau PET data would be acquired from the same individuals to determine antemortem versus postmortem correlations of tau burden in bvAD. Fourth, the inclusion of right hemispheric regions only in the postmortem evaluations may have created a bias. However, given the demonstrated right hemispheric dominance in bvFTD^39^ and suggested dominance in bvAD^3 5 27^ as well as established relationships between right frontal areas and behavioural deficits like apathy, disinhibition and aberrant motor behaviour,^40^ it is not likely that this affected our results. Fifth, the comparison of the frontal pole in the postmortem study against the medial and lateral frontal cortices in the tau PET study may introduce a bias, as these regions have been differentially implicated in behavioural disturbances.^41^ Sixth, although we did not specifically focus on the dysexecutive variant of AD in this study, executive deficits comprised one of the 6 core phenotypic inclusion criteria. Whereas the inclusion of 2/6 bvFTD symptoms strictly allows for inclusion based on one behavioural symptom in addition to executive dysfunction, all cases in our study had at least two behavioural features. Future studies should investigate the differences and overlap between dysexecutive and behavioural variants of AD. Seventh, questionnaires designed for bvFTD-like symptoms should be employed uniformly across cohorts, to quantify behavioural dysfunction and aid the diagnosis of bvAD.

Although the neurobiological mechanisms in bvAD are more similar to tAD than bvFTD, clinical differentiation between bvAD and bvFTD remains a diagnostic challenge. MRI and [^18^F]FDGPET provide only modest diagnostic accuracy^1 3^ and amyloid-β positivity on PET or CSF also occurs frequently in bvFTD patients, especially with advancing age and in the presence of an *APOE* ε4 allele.^42^ Tau PET, however, shows very high specificity for tau neurofibrillary tangles in AD dementia,^43^ as tau PET signal is low in non-AD neurodegenerative disorders (including sporadic forms of bvFTD^44^). The recent U.S. Food and Drug Administration approval of [^18^F]flortaucipir PET for clinical use may therefore aid the differential diagnosis between bvAD and bvFTD in clinical practice. Ultimately, clinical consensus criteria and standardisation of behavioural assessment are necessary to improve diagnosis, prognosis and patient care for individuals with bvAD.

## Data Availability

Data are available upon reasonable request. Anonymised data used in the present study may be available upon reasonable request to the corresponding author.
