# Supplementary material for: Heterogeneous distribution of tau pathology in the behavioural variant of Alzheimer’s disease
Source: J Neurol Neurosurg Psychiatry. 2021 Apr 13;92(8):872–80. doi: 10.1136/jnnp-2020-325497 (PMC8292599; doi:10.1136/jnnp-2020-325497)
Supplement: Supplementary data [file jnnp-2020-325497supp001.pdf]

Singleton et al.

SUPPLEMENTAL MATERIAL

Supplemental table 1. Clinical descriptions of bvAD cases

**ADC**	**bvAD case #1**<br>a right-handed individual in the age range of 70-75 years old, who reported little complaints himself, while his partner reported progressive behavioral changes in addition to memory deficits and slight word finding deficits, starting approximately 10 years ago. Upon cognitive evaluation, he showed a head turning sign and inappropriate disruption of conversation, and a euphoric affect, and obtained an MMSE score of 17/30. Neuropsychological testing showed deficits in speed of processing, memory and executive domains. The EEG was normal and largely influenced by drowsiness, and the MRI showed hippocampal atrophy (MTA 2/2), parietal atrophy (Koedam 1/1) and vascular damage (Fazekas 3). A lumbar punction was performed and showed an AD profile (Abeta1-42: 608, t-tau: 571, p-tau: 73), resulting in a differential diagnosis of possible FTD, with comorbid AD and vascular pathology. An FDG-PET scan was performed, showing strong abnormalities in bifrontal, lateral frontal, posterior cingulate and biparietal regions, with relative sparing of anterio-temporal regions, suggestive of AD or FTD. Genetic screening showed no autosomal dominant mutations and *APOE* ε3 homozygosity.	**bvAD case #2**<br>A right-handed individual in the age range of 60-65 years old, who reported mainly with complaints of dizziness, while his partner reported predominant and slowly progressive behavioral changes and paranoid thoughts, which have developed over the last 4 years and seemed to be an exacerbation of premorbid personality. Upon cognitive evaluation, the patient was found to be verbose in his speech, showing memory deficits and a preoccupation with his physical complaints. He showed a head turning sing, had an MMSE score of 27/30, a GDS score of 6/15 and a CDR score of 1. Neuropsychological testing showed deficits in speed of processing, memory and executive functioning. The EEG showed a light focal bitemporal abnormalities, the MRI showed slight biparietal atrophy (Koedam 1/1) and white matter abnormalities (Fazekas 1). Based on these findings, a differential diagnosis of bvFTD or AD was established. A [^{18}F]Florbetaben PET was performed and was rated as positive, leading to a diagnosis of AD with behavioral and psychotic symptoms. Genetic screening showed *APOE* ε3 homozygosity.	
**UCSF**	**bvAD case #3**<br>an ambidexter individual in the age range of 55-60 years old, presenting clinically with early and predominant behavioral changes. He obtained an MMSE score of 22, a CDR of 1 and	**bvAD case #4**<br>a left-handed individual in the age range of 55-60 years old presenting with predominant behavioral symptoms. He obtained an MMSE score of 21, and neuropsychological testing showed deficits in memory,	**bvAD case #5**<br>A left-handed individual in the age range of 75-80 years old presenting with predominant behavioral changes. He obtained an MMSE score of 19, a CDR of 2, and neuropsychological

Singleton E, *et al. J Neurol Neurosurg Psychiatry* 2021; 92:872–880. doi: 10.1136/jnnp-2020-325497

	neuropsychological testing showed deficits in memory and executive functioning. Genetic screening showed *APOE* ε3 homozogysity. A [^{18}F]Florbetaben-amyloid-PET was performed and rated positive, leading to a diagnosis of AD with behavioral features.	executive functioning and attention domains. Genetic screening showed an *APOE*-ε3ε4 profile. A [^{11}C]Pittsburgh Compound-B (PIB)-amyloid-PET was performed and was rated as positive, leading to a diagnosis of AD with behavioral features.	testing showed deficits in memory and executive functioning domains. A [^{11}C]Pittsburgh Compound-B (PIB)-amyloid-PET was performed and rated positive, leading to a diagnosis of AD with behavioral features.
**BioFINDER**	**bvAD case #6**<br>A right-handed individual in the age range of 75-80 years old, presenting with prominent behavioral changes. Neuropsychological testing revealed deficits in executive functioning, and relative sparing of memory and visuospatial skills. Genetic testing showed an *APOE*-ε3ε4 genotype. CSF showed an AD profile (Abeta1-42: 425, t-tau: 854, p-tau: 112, ratio AB42/40 0.35), leading to a diagnosis of AD with behavioral features.	**bvAD case #7**<br>A right-handed individual in the age range of 65-70 years old, presenting with predominant behavioral changes. Neuropsychological testing showed relative sparing of memory but not of visuospatial skills, and deficits in executive functioning. Genetic testing showed an *APOE*-ε3ε4 genotype. CSF showed an AD profile (AB42 452, t-tau 664, p-tau 82, ratio AB42/40 0,52), leading to a diagnosis of AD with behavioral features.	

Singleton E, *et al. J Neurol Neurosurg Psychiatry* 2021; 92:872–880. doi: 10.1136/jnnp-2020-325497

Supplemental table 2. PET acquisition and processing details per center.

Center	Tracer, time frame, reference region & atlas	Acquisition details	Image processing details
**ADC**	[^{18}F]Flortaucipir, 80-100 min postinjection, cerebellar gray matter, Hammers & Svarer atlas	All structural whole-brain MRI scans were performed on a single 3.0-T Philips Ingenuity Time-of-Flight PET/MR scanner (Philips Medical Systems, Best, The Netherlands). Isotropic structural 3D T1-weightead images were acquired using a sagittal turbo gradient-echo sequence (1.00mm3 isotropic voxels, repetition time = 7.9ms, echo time = 4.5 ms, and flip angle = 8°). Tau PET images were acquired on a PET/CT scanner (Ingenuity TF ’13 [n = 41], Ingenuity TF ’17 [n = 18], Philips Medical Systems, Best, The Netherlands). Individual doses of [^{18}F]flortaucipir were prepared on site in accordance with Avid Radiopharmaceuticals quality control release criteria[1]. Head movements were restricted by using a headband. During scan procedures, head movement was monitored using laser beams and, if necessary, corrected. After a low-dose CT for attenuation correction, 233.26 ± 12.87 MBq [^{18}F]flortaucipir was injected (injected mass 1.25 ± 0.96 µg) simultaneously with the start of a 60-min dynamic emission scan. After a 20-min break and a second low-dose CT, a second dynamic emission scan was started from 80 to 130 min post injection. List mode data were reconstructed using 3D RAMLA with a matrix size of 128 × 128 × 90 and a voxel size of 2 × 2 × 2 mm^3. The second scan session was co-registered to the first, and the two parts were combined into a single data set of 29 frames using an in-house developed code consisting of a multi-frame co-registration feature of Vinci implemented in IDL. Standard corrections (using Philips Healthcare software) for dead time, decay, attenuation, randoms, and scatter were performed[1].	T1-weighted MR images were co-registered to their individual PET scans in native space using Vinci software (Max Plank Institute, Cologne, Germany). The Hammers template[2], incorporated in PVElab software, was used to delineate cerebellar gray matter region of interest (ROI) and other ROIs on the co-registered MR images. For the entorhinal cortex, the Svarer atlas was used[3]. Standardized uptake value ratios (SUVR) images were obtained using the cerebellar gray matter ROI obtained from the Hammers template as a reference region using the 80-100 min time interval postinjection. For visual comparisons, all co-registered T1-weighted MRI scans were warped to Montreal Neurological Institute (MNI152) space using SPM12, and these transformation matrixes were used to warp native space SUVR images to MNI space. All warped images were checked manually for transformation errors. Next, the normalized PET images were smoothed using an 8-mm Gaussian kernel. As a comparison to the PET images of the bvAD cases, we created average SUVR images for the typical AD groups in each center.
**UCSF**	[^{18}F]Flortaucipir, 80-100 min postinjection, inferior cerebellar gray matter, Desikan-Killiany atlas	PET scans were performed at LBNL on a Siemens Biograph 6 Truepoint PET/CT scanner in 3D acquisition mode. A low-dose CT/transmission scan was performed for attenuation correction prior to all scans. [^{18}F]Flortaucipir was synthesized and radiolabelled at LBNL’s Biomedical Isotope Facility.	PET images were co-registered to the subjects’ MP-RAGE using Statistical Parametric Mapping (SPM) version 8 (Wellcome Trust Centre for Neuroimaging, Institute of Neurology at University College London). SUVR images at t = 80–100 minutes post-injection were created by normalizing summed

		Approximately 10 mCi of [18F]Flortaucipir was injected intravenously, and subjects participated in one of two acquisition schemes: 0–100-min postinjection full dynamic scans (4 x 15, 8 x 30, 9 x 60 s, and 2 x 3, 16 x 5 min frames) followed by 120–150 min (6 x 5 min frames), or 75–115 min (8 x 5 min frames). PET data were reconstructed using an ordered subset expectation maximization algorithm with weighted attenuation. Images were smoothed with a 4mm Gaussian kernel with scatter correction and evaluated prior to analysis for patient motion and adequacy of statistical counts.	activity from the realigned frames to mean activity in cerebellar grey matter. Images were smoothed using a 8 mm Gaussian kernel. For the typical AD comparison, average SUVR images were created for the typical AD subjects.
**BioFINDER**	[18F]RO948, 70-90 min postinjection, inferior cerebellar gray matter, Desikan Killiany atlas	All study participants underwent two PET scans on a digital GE Discovery MI scanner (General Electric Medical Systems). Participants were injected with 365 ± 20 MBq of [18F]RO948, and LIST mode emission data was acquired for each scan of 70–90 min ([18F]RO948) post injection. Low-dose CT scans were performed immediately prior to the PETscans for attenuation correction. PET data was reconstructed using VPFX-S (ordered subset expectation maximization (OSEM) with time-of-flight (TOF) and point spread function (PSF) corrections) with 6 iterations and 17 subsets with 3 mm smoothing, standard Z filter, and 25.6-cm field of view with a 256 × 256 matrix. LIST mode data was binned into 4 × 5-min time frames, and the resulting PET images motion corrected, summed, and co-registered to their corresponding T1-weighted MR images.	ROIs were based on the parcellation of the T1-weighted MRI using FreeSurfer v6.0 (https://surfer.nmr.mgh.harvard.edu/). Standardized uptake value ratio (SUVR) images were calculated using an inferior cerebellar reference region[4]. SUVR PET images were spatially transformed into a common MNI152 space using the transformation derived from MRI normalization and smoothed at 6 mm with a full width at half maximum Gaussian kernel. Calculations were performed using SPM12 (Wellcome Department of Cognitive Neurology, London, UK; http://www.fil.ion.ucl.ac.uk/spm) in MATLAB (v. 9.2, 2017a). Images were smoothed using a 8 mm Gaussian kernel. For comparison purposes, average SUVR image was created of the typical AD group. Composite ROIs were created for several regions.

Singleton E, *et al. J Neurol Neurosurg Psychiatry* 2021; 92:872–880. doi: 10.1136/jnnp-2020-325497

Supplemental table 3. Overview of individual regions within the composite regions-of-interest used for the PET analyses.

Center	Atlas	Composite region	Subregion codes
**ADC**	Svarer	Entorhinal cortex	L_entorhinc, R_entorhinc
	Hammers (all other regions than entorhinal cx)	Temporoparietal cortex	Left_Superior_temporal_gyrus, Right_Superior_temporal_gyrus, Left_Middle_and_inferior_temporal_gyri, Right_Middle_and_inferior_temporal_gyri, Left_Inferolateral_remainder_of_parietal_lobe, Right_Inferolateral_remainder_of_parietal_lobe, Left_Superior_parietal_gyrus, Right_Superior_parietal_gyrus, Left_Gyrus_cinguli_posterior_part, Right_Gyrus_cinguli_posterior_part
		Frontal cortex	Left_Middle_frontal_gyrus, Right_Middle_frontal_gyrus, Left_Orbitofrontal_gyri, Right_Orbitofrontal_gyri, Left_Superior_frontal_gyrus, Right_Superior_frontal_gyrus, Left_Inferior_frontal_gyrus, Right_Inferior_frontal_gyrus, Left_Gyrus_rectus, Right_Gyrus_rectus
		Insular cortex	Left_Insula, Right_Insula
		Mean cortical ROI	Left_Gyrus_rectus, Right_Gyrus_rectus, Left_Middle_frontal_gyrus, Right_Middle_frontal_gyrus, Left_Orbitofrontal_gyri, Right_Orbitofrontal_gyri, Left_Inferior_frontal_gyrus, Right_Inferior_frontal_gyrus, Left_Superior_frontal_gyrus, Right_Superior_frontal_gyrus, Left_Lateral_remainder_of_occipital_lobe, Right_Lateral_remainder_of_occipital_lobe, Left_Lingual_gyrus, Right_Lingual_gyrus, Left_Cuneus, Right_Cuneus, Left_Entorhinal, Right_Entorhinal, Right_Anterior_temporal_lobe_lateral_part, Left_Anterior_temporal_lobe_lateral_part, Right_Anterior_temporal_lobe_medial_part, Left_Anterior_temporal_lobe_medial_part, Right_Superior_temporal_gyrus, Left_Superior_temporal_gyrus, Right_Middle_and_inferior_temporal_gyri, Left_Middle_and_inferior_temporal_gyri, Right_Posterior_temporal_lobe, Left_Posterior_temporal_lobe, Right_Fusiform_gyrus, Left_Fusiform_gyrus, Left_Inferolateral_remainder_of_parietal_lobe, Right_Inferolateral_remainder_of_parietal_lobe, Left_Superior_parietal_gyrus, Right_Superior_parietal_gyrus, Left_Gyrus_cinguli_posterior_part, Right_Gyrus_cinguli_posterior_part, Left_Gyrus_cinguli_anterior_part, Right_Gyrus_cinguli_anterior_part, Left_Insula, Right_Insula
**UCSF**	Desikan-Killiany	Entorhinal cortex	lhentorhinal, rhentorhinal
		Temporoparietal cortex	lhsuperiortemporal, rhsuperiortemporal, lhmiddletemporal, rhmiddletemporal, lhinferiortemporal, rhinferiortemporal, lhtransversetemporal, rhtransversetemporal, lhbankssts, rhbankssts, lhsupramarginal, rhsupramarginal, lhsuperiorparietal, rhsuperiorparietal, lhinferiorparietal, rhinferiorparietal, lhprecuneus, rhprecuneus, lhisthmuscingulate, rhisthmuscingulate
		Frontal cortex	lhsuperiorfrontal, rhsuperiorfrontal, lhrostralmiddlefrontal, rhrostralmiddlefrontal, lhcaudalmiddlefrontal, rhcaudalmiddlefrontal, lhparsopercularis, rhparsopercularis, lhparstriangularis, rhparstriangularis, lhparsorbitalis, rhparsorbitalis, lhlateralorbitofrontal, rhlateralorbitofrontal, lhmedialorbitofrontal, rhmedialorbitofrontal, lhfrontalpole, rhfrontalpole
		Insular cortex	lhinsula, rhinsula

		Mean cortical ROI	lhentorhinal, rhentorhinal, lhsuperiortemporal, rhsuperiortemporal, lhmiddletemporal, rhmiddletemporal, lhinferiortemporal, rhinferiortemporal, lhtransversetemporal, rhtransversetemporal, lhbankssts, rhbankssts, lhsupramarginal, rhsupramarginal, lhsuperiorparietal, rhsuperiorparietal, lhinferiorparietal, rhinferiorparietal, lhprecuneus, rhprecuneus, lhisthmuscingulate, rhisthmuscingulate, lhposteriorcingulate, rhposteriorcingulate, lhlingual, rhlingual, lhpericalcarine, rhpericalcarine, lhcuneus, rhcuneus, lhlateraloccipital, rhlateraloccipital, lhsuperiorfrontal, rhsuperiorfrontal, lhrostralmiddlefrontal, rhrostralmiddlefrontal, lhcaudalmiddlefrontal, rhcaudalmiddlefrontal, lhparsopercularis, rhparsopercularis, lhparstriangularis, rhparstriangularis, lhparsorbitalis, rhparsorbitalis, lhlateralorbitofrontal, rhlateralorbitofrontal, lhmedialorbitofrontal, rhmedialorbitofrontal, lhfrontalpole, rhfrontalpole, lhtemporalpole, rhtemporalpole, lhrostralanteriorcingulate, rhrostralanteriorcingulate, lhcaudalanteriorcingulate, rhcaudalanteriorcingulate, lhinsula, rhinsula
**BioFINDER**	Desikan-Killiany	Entorhinal cortex	lhentorhinal, rhentorhinal
		Temporoparietal cortex	lhsuperiortemporal, rhsuperiortemporal, lhmiddletemporal, rhmiddletemporal, lhinferiortemporal, rhinferiortemporal, lhtransversetemporal, rhtransversetemporal, lhbankssts, rhbankssts, lhsupramarginal, rhsupramarginal, lhsuperiorparietal, rhsuperiorparietal, lhinferiorparietal, rhinferiorparietal, lhprecuneus, rhprecuneus, lhisthmuscingulate, rhisthmuscingulate
		Frontal cortex	lhsuperiorfrontal, rhsuperiorfrontal, lhrostralmiddlefrontal, rhrostralmiddlefrontal, lhcaudalmiddlefrontal, rhcaudalmiddlefrontal, lhparsopercularis, rhparsopercularis, lhparstriangularis, rhparstriangularis, lhparsorbitalis, rhparsorbitalis, lhlateralorbitofrontal, rhlateralorbitofrontal, lhmedialorbitofrontal, rhmedialorbitofrontal, lhfrontalpole, rhfrontalpole
		Insular cortex	lhinsula, rhinsula
		Mean cortical ROI	lhentorhinal, rhentorhinal, lhsuperiortemporal, rhsuperiortemporal, lhmiddletemporal, rhmiddletemporal, lhinferiortemporal, rhinferiortemporal, lhtransversetemporal, rhtransversetemporal, lhbankssts, rhbankssts, lhsupramarginal, rhsupramarginal, lhsuperiorparietal, rhsuperiorparietal, lhinferiorparietal, rhinferiorparietal, lhprecuneus, rhprecuneus, lhisthmuscingulate, rhisthmuscingulate, lhposteriorcingulate, rhposteriorcingulate, lhlingual, rhlingual, lhpericalcarine, rhpericalcarine, lhcuneus, rhcuneus, lhlateraloccipital, rhlateraloccipital, lhsuperiorfrontal, rhsuperiorfrontal, lhrostralmiddlefrontal, rhrostralmiddlefrontal, lhcaudalmiddlefrontal, rhcaudalmiddlefrontal, lhparsopercularis, rhparsopercularis, lhparstriangularis, rhparstriangularis, lhparsorbitalis, rhparsorbitalis, lhlateralorbitofrontal, rhlateralorbitofrontal, lhmedialorbitofrontal, rhmedialorbitofrontal, lhfrontalpole, rhfrontalpole, lhtemporalpole, rhtemporalpole, lhinsula, rhinsula, lhrostralanteriorcingulate, rhrostralanteriorcingulate, lhcaudalanteriorcingulate, rhcaudalanteriorcingulate

Supplemental table 4. Details of neuropathological methods.

Center	Methods
Amsterdam UMC – location VUmc	First, formalin fixated brain regions were embedded in paraffin, cut into 8 μm thick sections and mounted onto SuperFrost Plus glass slides (Menzel-Gläser, Braunschweig, Germany). The immunostaining of all included slides was performed in one batch to minimize experimental variability. Slides were deparaffinised and rehydrated by taking them through a xylene to alcohol series. Heat-induced antigen retrieval was performed in citrate buffer (pH 6) using an autoclave (120 °C for 5 min). Endogenous peroxidase activity was blocked by incubation in 0.3% H_2O_2 in phosphate buffer saline (PBS; pH 7.4) for 30 min. Then, slides were incubated in primary antibody diluent (AT8) for 1 hour at room temperature, followed by an incubation in HRP-labelled EnVision (K5007; DAKO, Glostrup, Denmark) for 30 min. Between steps, slides were washed with PBS (3×5 min). Visualization of immunostaining was attained with chromogen 3.3'-diaminobenzidine (DAB: K5007; DAKO). Slides were counterstained with Mayers Haematoxylin, dehydrated by taking them through an alcohol to xylene series and then cover slipped with Quick-D (Klinipath, Duiven, The Netherlands). In each brain region, two ROIs were randomly selected and photographed. Images were taken using a x4 objective for cortices and a x10 objective for subcortical areas on an Olympus BX 41 microscope with Leica MC 170 HD digital camera (2,592x194 pixels). For cortical regions the area between the sulci and crown of the gyrus was taken where all six cortical layers run parallel to the pia. Within the image, ROIs were drawn manually to include cortical tissue only, and therefore the total surface varied for each ROI as this was dependent on regional cortical atrophy.

Singleton E, *et al. J Neurol Neurosurg Psychiatry* 2021; 92:872–880. doi: 10.1136/jnnp-2020-325497

BMJ Publishing Group Limited (BMJ) disclaims all liability and responsibility arising from any reliance placed on this supplemental material which has been supplied by the author(s)

Supplemental table 5. Regional W-scores of tau SUVR in participants with bvAD (presented individually) versus participants with typical AD (presented as group average (SD)[lower and upper bound]), corrected for age, sex and MMSE.

	**ADC**			**UCSF**				**BioFINDER**		
	**bvAD$_1$**	**bvAD$_2$**	**Typical AD (n=55)**	**bvAD$_3$**	**bvAD$_4$**	**bvAD$_5$**	**Typical AD (n=50)**	**bvAD$_6$**	**bvAD$_7$**	**Typical AD**
Entorhinal cortex	-0.61428	1.50544	0.00011 (1) [-2.06290-2.38031]	-0.03040	-0.24040	0.74930	0.00011 (1) [-2.06290-2.38031]	-0.10117	-0.01877	-0.00070 (1 [-3.27755-2
Temporoparietal cortex	0.45435	0.54546	0.00057 (1) [-2.83479-2.17616]	-0.43327	-0.30644	0.55797	0.00057 (1) [-2.83479-2.17616]	0.77621	1.15157	0.00041 (1) [-2.52419-2
Frontal cortex	0.85427	1.93316	-0.00035 (1) [-2.31959-2.66509]	-0.49199	0.00457	1.68456	-0.00035 (1) [-2.31959-2.66509]	0.79069	1.47207	-0.00097 (1 [-1.77143-5
Medial Prefrontal cortex	0.80862	1.22752	-0.00103 (1) [-2.04051-2.52739]	-0.23570	0.06020	2.12807	-0.00103 (1) [-2.04051-2.52739]	0.77491	1.12047	-0.00023 (1 [-1.80160-5
Lateral Prefrontal cortex	0.86863	2.79360	0.00101 (1) [-2.65760-2.71078]	-0.70860	-0.05853	1.22578	0.00101 (1) [-2.65760-2.71078]	0.79563	1.77703	-0.00061 (1 [-1.71568-5
Insula	1.47487	0.21200	-0.00015 (1) [-1.94050-2.27901]	-0.60329	-0.54791	1.30190	-0.00015 (1) [-1.94050-2.27901]	0.40432	-0.07945	0.00002 (1) [-2.31365-3
Mean cortical	0.66773	0.64005	0.00054 (1) [-2.84334-2.26661]	-0.45077	-0.34003	1.14819	0.00054 (1) [-2.84334-2.26661]	0.75740	1.33167	0.00064 (1 [-2.55094-3
Ratio Frontal:Entorhinal	1.36210	0.33047	0.00015 (1) [-2.13332-2.50381]	-0.49660	0.34730	1.02413	0.00015 (1) [-2.13332-2.50381]	0.88832	2.04024	0.00007 (1) [-1.76568-4
Ratio Frontal:Parietal	0.98750	0.84131	-0.00011 (1) [-2.22511-3.02359]	-0.20583	0.36249	1.46388	-0.00011 (1) [-2.22511-3.02359]	0.12272	0.44605	0.00005 (1) [-1.65837-4

Singleton E, *et al. J Neurol Neurosurg Psychiatry* 2021; 92:872–880. doi: 10.1136/jnnp-2020-325497

BMJ Publishing Group Limited (BMJ) disclaims all liability and responsibility arising from any reliance placed on this supplemental material which has been supplied by the author(s)

Supplemental table 6. Tau SUVR within network templates in participants with bvAD (presented individually) versus participants with typical AD (presented as group average (SD)[lower and upper bound]).

	**ADC**			**UCSF**				**BioFINDER**		
	**bvAD$_1$**	**bvAD$_2$**	**Typical AD (n=55)**	**bvAD$_3$**	**bvAD$_4$**	**bvAD$_5$**	**Typical AD (n=60)**	**bvAD$_6$**	**bvAD$_7$**	**Typical AD (n=90)**
Executive Control Network	1.68	2.20	1.59 (0.41) [0.79-2.44]	1.64	1.95	1.96	1.96 (0.65) [0.89-3.38]	1.35	2.44	1.69 (0.65) [0.63-4.20]
Anterior Salience Network	1.46	1.95	1.24 (0.30) [0.68-2.16]	1.35	1.62	2.06	1.61 (0.47) [0.89-2.71]	1.19	1.88	1.37 (0.61) [0.63-4.62]
Anterior Default Mode Network	1.21	1.31	1.17 (0.22) [0.72-1.87]	1.09	1.49	2.31	1.43 (0.33) [0.99-2.40]	1.03	1.27	1.25 (0.50) [0.61-4.46]
Posterior Default Mode Network	1.49	1.95	1.64 (0.46) [0.82-2.67]	2.02	2.19	2.29	2.25 (0.80) [1.02-4.12]	1.47	2.37	1.86 (0.68) [0.73-3.89]

Singleton E, *et al. J Neurol Neurosurg Psychiatry* 2021; 92:872–880. doi: 10.1136/jnnp-2020-325497

Supplemental table 7. W-scores of tau SUVR within network templates in participants with bvAD (presented individually) versus participants with typical AD (presented as group average (SD)[lower and upper bound]), corrected for age, sex and MMSE.

	**ADC**			**UCSF**				**BioFINDER**		
	**bvAD$_1$**	**bvAD$_2$**	**Typical AD (n=55)**	**bvAD$_3$**	**bvAD$_4$**	**bvAD$_5$**	**Typical AD (n=50)**	**bvAD$_6$**	**bvAD$_7$**	**Typical AD (n=90)**
Executive Control Network	0.82405	1.88733	-0.00035 (1) [-3.20837-2.57066]	-0.53796	0.03513	0.83100	-0.00035 (1) [-2.47742- 2.14928]	1.00266	1.72788	-0.00034 (1) [-2.39726-3.73957]
Anterior Salience Network	1.05694	2.77345	-0.00070 (1) [-2.09140-2.67840]	-0.73858	-0.08946	1.69940	-0.00023 (1) [–2.10627-3.19533]	0.84409	1.15080	0.00064 (1) [-1.62012-4.49929]
Anterior Default Mode Network	0.31385	0.82503	-0.00017 (1) [-1.71209-3.32222]	-1.47521	0.11788	3.79406	-0.00009 (1) [-1.89114-2.27975]	0.54982	0.25916	-0.000 (1) [-1.66231-5.75619]
Posterior Default Mode Network	0.21462	0.65858	-0.00033 (1) [-3.18177-1.78722]	-0.30160	-0.05143	1.18150	0.00033 (1) [-2.88946-1.97586]	0.89286	1.16524	-0.00015 (1) [-2.45116-3.01871]

Supplemental table 8. Percentage of tau pathology in several regions of interest according to patient group.

	**bvAD**	**Typical AD**	**P-value**
n	8	7	
ACC[a]	41.66 (9.58)	40.50 (12.81)	0.125
Hippocampus CA1[b]	42.59 (6.87)	39.02 (4.63)	0.433
Caudate nucleus	16.87 (14.70)	18.88 (13.28)	0.195
Entorhinal cortex[c]	44.55 (3.85)	41.85 (7.73)	0.948
Frontal pole[d]	45.01 (8.93)	43.57 (5.16)	0.693
Fronto-Insula	36.38 (18.87)	48.76 (4.46)	0.337
Putamen	4.39 (4.62)	16.22 (14.08)	0.121
Subiculum[e]	50.77 (4.97)	40.97 (5.55)	0.111
Thalamus[f]	3.74 (3.00)	4.64 (3.39)	0.805
Ratio F:E[g]	1.08 (0.08)	1.15 (0.24)	0.992
Ratio F:H[h]	1.22 (0.24)	1.14 (0.20)	0.642

P-values represent results from univariate ANCOVAs on the ranks of each region, correcting for age and sex.

bvAD = behavioral variant of AD, *ACC* = anterior cingulate cortex, *Ratio F:E* = ratio frontal-to-entorhinal tau, *Ratio F:H* = ratio frontal-to-hippocampal tau.

[a]n=8 for bvAD, n=5 for typical AD, [b]n=4 for bvAD, n=6 for typical AD, [c]n=4 for bvAD, n=2 for typical AD, [d]n=5 for bvAD, n=5 for typical AD, [e]n=4 for bvAD, n=6 for typical AD, [f]n=8 for bvAD, n=6 for typical AD, [g]n=3 for bvAD, n=4 for typical AD, [h]n=3 for bvAD, n=4 for typical AD.

SUPPLEMENTAL REFERENCES

1. Golla SSV, Timmers T, Ossenkoppele R, et al. Quantification of Tau Load Using [(18)F]AV1451 PET. Molecular imaging and biology 2017;19:963-971.

2. Hammers A, Allom R, Koepp MJ, et al. Three-dimensional maximum probability atlas of the human brain, with particular reference to the temporal lobe. Human brain mapping 2003;19:224-247.

3. Svarer C, Madsen K, Hasselbalch SG, et al. MR-based automatic delineation of volumes of interest in human brain PET images using probability maps. NeuroImage 2005;24:969-979.

4. Baker SL, Maass A, Jagust WJ. Considerations and code for partial volume correcting [(18)F]-AV-1451 tau PET data. Data in brief 2017;15:648-657.
